# Genomic Features and Antimicrobial Activity of *Phaeobacter inhibens* Strains from Marine Biofilms

**DOI:** 10.3390/md22110492

**Published:** 2024-10-31

**Authors:** Han Cui, Jie Lu, Wei Ding, Weipeng Zhang

**Affiliations:** 1 MOE Key Laboratory of Evolution & Marine Biodiversity, Institute of Evolution & Marine Biodiversity, Ocean University of China, Qingdao 266003, China; cuihan@stu.ouc.edu.cn (H.C.); lujie6636@stu.ouc.edu.cn (J.L.); 2 MOE Key Laboratory of Marine Genetics & Breeding, College of Marine Life Sciences, Ocean University of China, Qingdao 266003, China; dingwei@ouc.edu.cn

**Keywords:** marine biofilms, *Phaeobacter inhibens*, antimicrobial activity

## Abstract

Members of the genus *Phaeobacter* are widely distributed in the marine environment and are known for their ability to produce tropodithietic acid (TDA). Studies investigating the genomic and metabolic features of *Phaeobacter* strains from marine biofilms are sparse. Here, we analyze the complete genomes of 18 *Phaeobacter* strains isolated from biofilms on subtidal stones, with the aim of determining their potential to synthesize secondary metabolites. Based on whole-genome comparison and average nucleotide identity calculation, the isolated bacteria are classified as novel strains of *Phaeobacter inhibens*. Further analysis reveals a total of 153 biosynthetic gene clusters, which are assigned to 32 gene cluster families with low similarity to previously published ones. Complete TDA clusters are identified in 14 of the 18 strains, while in the other 4 strains the TDA clusters are rather incomplete and scattered across different chromosome and plasmid locations. Phylogenetic analysis suggests that their presence or absence may be potentially attributed to horizontal gene transfer. High-performance liquid chromatography–mass spectrometry analysis demonstrates the production of TDA in all the examined strains. Furthermore, the *Phaeobacter* strains have strong antibacterial activity against the pathogenic strain *Vibrio owensii* ems001, which is associated with acute hepatopancreatic necrosis in South American white shrimp. Altogether, this study ameliorates our knowledge of marine biofilm-associated *Phaeobacter* and offers new avenues for exploiting marine antimicrobial agents.

## 1. Introduction

Marine bacteria are known to produce a variety of secondary metabolites with significant biological activities [1]. The natural products produced by these organisms deem them a rich source of novel bioactive secondary metabolites for drug discovery [2,3]. The genus *Phaeobacter* belongs to the marine *Roseobacteraceae* family (previously known as *Roseobacter* clade and belonging to alphaproteobacteria). First isolated from the German Wadden Sea [4], members of *Phaeobacter* are typically heterotrophic and play significant roles in marine ecosystems [5]. These bacteria have gained scientific interest owing to their ability to produce a potent antibacterial compound, named tropodithietic acid (TDA) [6,7]. TDA is a sulfur-containing tropone derivative [8], and it is known that *tdaA* (the LysR family transcriptional regulator), *tdaB* (glutathione S-transferase), *tdaC* (prephenate dehydratase), *tdaD* (the thioesterase-like superfamily), *tdaE* (acyl-CoA dehydrogenase), and *tdaF* (flavoprotein) are involved in the biosynthesis of TDA [9,10,11,12,13]. For example, the LysR family transcriptional regulator, *tdaA*, is a positive regulator of the expression of other genes involved in TDA synthesis and binds to the *tda* promoter region [10]. Another core gene, *tdaB*, encodes glutathione S-transferase, which belongs to the bacterial GST protein family and plays a catalytic role in the conversion of S-thiocysteine to CoA ester [11]. The gene *tdaE* is involved in the metabolism, addition, and modification of CoA, as well as the oxidation reaction [9,12,13]. TDA can inhibit the growth of bacterial pathogens in fish and human beings, as well as many non-pathogenic bacteria [14,15,16,17,18,19]. For instance, TDA prevents vibriosis in cod larvae challenged with *Vibrio anguillarum* [20]. In addition, TDA serves as a signaling molecule that enables quorum sensing and regulates the behavior of *Phaeobacter inhibens* [21]. 

While research studies have been conducted on marine *Roseobacteraceae*, the strains of marine biofilm-associated *Phaeobacter* have not been well studied. Biofilms are one of the most widespread and successful life forms on Earth and are now considered as the primary mode of microbial life [22]. In marine environments, biofilms often develop on man-made artificial objects and biological surfaces such as artificial panels [23,24,25], animal guts [26,27], microplastics [28,29], and stone surfaces [30,31]. A global survey stated that marine biofilms constitute over 7300 species and 11 million protein-coding genes that are otherwise undetectable in seawater, suggestive of the high potential of biofilms in the discovery of novel species and functions [32,33]. In our recent study [34], we identified *Roseobacteraceae* as one of the dominant bacterial groups in marine biofilms that exhibits unique methods of energy metabolism. Notably, the 54 *Roseobacteraceae* strains isolated from coastal marine biofilms and analyzed in our study included 18 strains of *P. inhibens*, which require in-depth analysis and characterization in terms of their genomic and metabolic features [34]. 

In the present study, we analyzed the complete genomes of the 18 *P. inhibens* strains isolated from coastal marine biofilms in terms of their strain-level diversity, genomic features, and secondary metabolite biosynthesis abilities. We also evaluated their ability to produce TDA using high-performance liquid chromatography (HPLC) coupled with mass spectrometry (MS). Furthermore, we tested their antibacterial activity against a pathogenic *Vibrio owensii* strain, a causative agent of acute hepatopancreatic necrosis (AHPND) in South American white shrimps. 

## 2. Results

### 2.1. Cell Phenotype and Genome Identity

**Figure 1:**
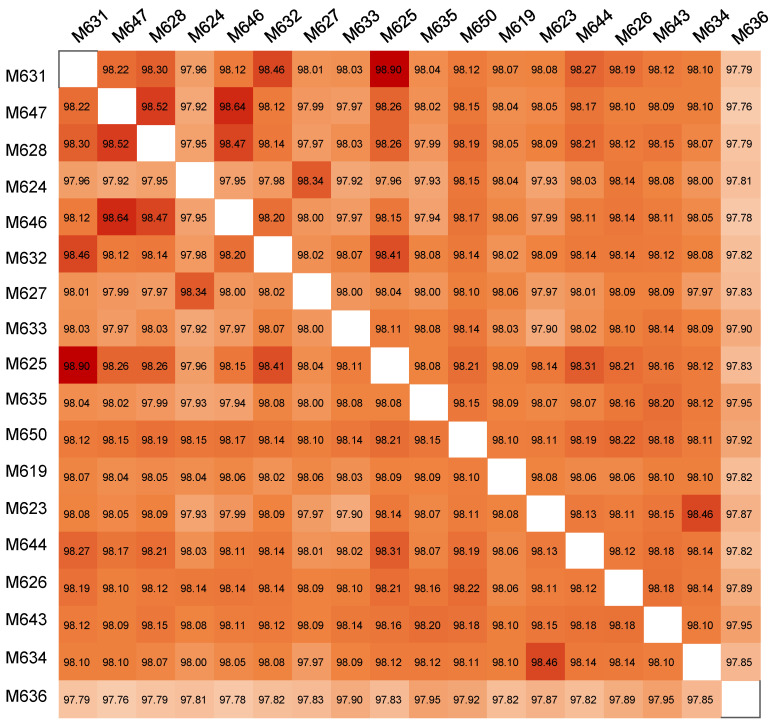
Comparison of pairwise average nucleotide identity among the 18 *Phaeobacter inhibens* genomes.

The phenotypes of the 18 *P. inhibens* strains were studied by transmission electron microscopy (TEM), which revealed similar cell sizes and shapes. The image of *P. inhibens* M619, a representative strain, is shown in Figure S1. The two cells in the field were elliptical in shape, with a long axis of approximately 1500 nm and a short axis of approximately 840 nm (Figure S1). The TEM images of the cells also revealed the production of extracellular matrices (Figure S1), implying their ability to form biofilms. Physiologically, these strains showed robust growth abilities in marine broth 2216E medium and achieved substantial cell biomass after being cultured overnight. Their genomic DNA was extracted for sequencing and to obtain complete genomes, which ranged in size from 4,089,150 to 4,402,211 bp (Table S1). GTDB-Tk analysis confirmed that all the strains belong to *P. inhibens.* Their genomes comprised a single chromosome and 3–5 plasmids (Table S1). The genomes displayed very similar GC contents (59.78% to 59.96%) and numbers of rRNA (*n* = 12) and tRNA (58 ≤ *n* ≤ 60) coding genes (Table S1). The number of predicted open-reading frames (ORFs) ranged from 3828 to 4167, which suggests considerable variations in terms of functional composition. The ORFs between 2245 and 2354 could be annotated by searching against the Kyoto Encyclopedia of Genes and Genomes (KEGG) database (Table S2). In addition, pairwise comparisons of average nucleotide identity (ANI) revealed values from 97.76% to 98.90% (Figure 1), indicating that these are distinct strains within the species *P. inhibens*.

### 2.2. Pangenome Analyses

**Figure 2:**
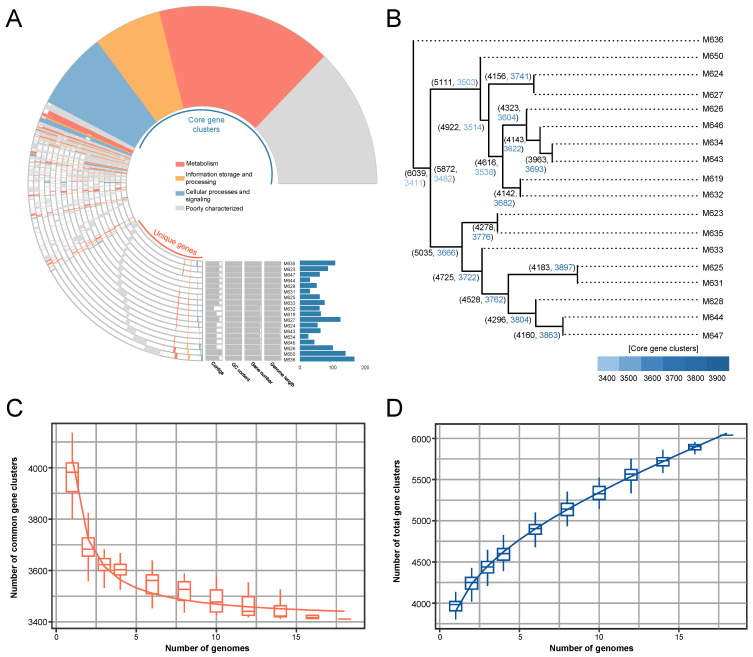
Pangenome analysis. (**A**) The pangenome profile displaying the functional distribution of core gene clusters alongside unique genes. (**B**) The total number of gene clusters and the number of core gene clusters of 18 strains on each branch displayed through a single nucleotide polymorphism (SNP)-based tree. (**C**) An accumulative curve depicting the relationship between the number of core gene clusters and the number of genomes. (**D**) An accumulative curve depicting the relationship between the total number of gene clusters and the number of genomes.

We conducted pangenome analysis using integrated prokaryotes genome and pan-genome analysis (IPGA) to investigate the core and unique genes among *P. inhibens* strains. Accordingly, 6093 orthologous genes were identified across the 18 genomes studied (Figure 2A). The results of the clustering analysis revealed a substantial number of strain-specific genes. For instance, the strain M636 shared the fewest genes (56.48%) with other strains and exhibited 167 unique genes, while M634 shared the greatest number of genes (93.18%) with other strains and carried 26 unique genes (Figure 2A). The core gene clusters primarily comprised genes that were involved in processes such as metabolism, information storage and processing, cellular processes, and signaling (Figure 2A). To visualize the relationships between these strains, a phylogenetic tree was constructed based on single nucleotide polymorphisms (SNPs), which categorized the strains into three groups (Figure 2B). Notably, M636 was classified into an independent branch from the other 17 strains (Figure 2B), consistent with the ANI results (Figure 1). Further, based on the accumulative curves of core gene clusters and the number of genomes, the number of core gene clusters eventually decreased from over 4000 to 3400 (Figure 2C). Thus, there exist genomic content variations in terms of gene clusters. Consistently, the number of all gene clusters possessed by the genomes increased from 4000 to 6000 once 2 to 18 genomes were included in analyses (Figure 2D).

### 2.3. Diversity and Evolution of Biosynthetic Gene Clusters (BGCs)

**Figure 3:**
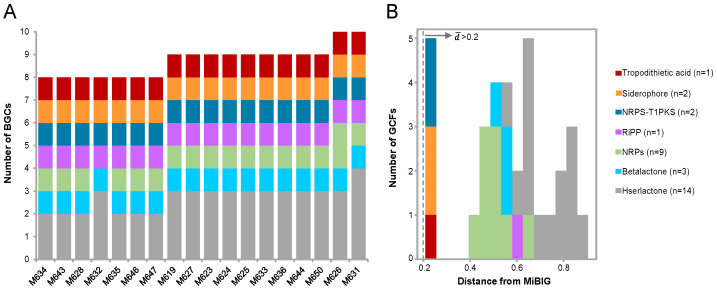
The summary and distribution of biosynthetic gene clusters (BGCs). (**A**) The distribution of the seven types of BGCs among the genomes. (**B**) The gene cluster family (GCF) distance from those in the Minimum Information about a Biosynthetic Gene cluster database (MIBiG). The number of GCFs in each category is shown in brackets.

The aforementioned findings indicate the considerable strain-to-strain variations in terms of their genome content. Next, we sought to predict the BGCs among the 18 genomes using antiSMASH. In total, 157 BGCs were identified, with an average of eight BGCs per genome. These BGCs were grouped as siderophore, post-translationally modified peptides (RiPPs), nonribosomal peptides (NRPs), NRPS-T1PKS compounds (hybrid compounds), betalactone, hserlactone, and others (Figure 3A). The "others" were manually annotated as tropodithietic acids, as stated in the next paragraph. To reveal the types of the BGCs, they were further classified into gene cluster families (GCFs). As such, 32 GCFs were identified across the 18 genomes, with an average of approximately 2 GCFs per genome (Figure 3B). These GCFs predominantly comprised 14 hserlactones and 9 NPRs (Figure 3B), indicating high diversity in these two groups. The RiPPs from these 18 strains were clustered into the same GCF, and the core biosynthetic genes of this RiPP GCF are DUF692 enzymes, known to mediate the chemical transformations leading to macrolides and heterocycles [35,36]. To evaluate their novelty, all the GCFs were compared with those recorded in the Minimum Information about a Biosynthetic Gene Cluster (MIBiG) database. The minimum cosine distances for all GCFs were greater than the threshold of 0.2 (Figure 3B), indicating high levels of novelty.

**Figure 4:**
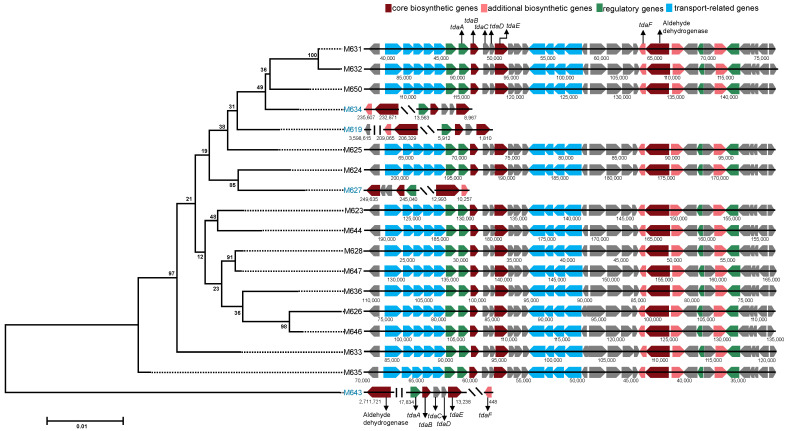
The evolution and gene sequential patterns of tropodithietic acid (TDA) BGCs. Left: the maximum likelihood phylogenetic tree constructed based on the core genes of TDA biosynthesis. Right: the organization of various gene types within the TDA BGCs, with distinct colors representing different gene categories.

We next focused on the BGCs of TDA. Based on the results of antiSMASH, complete TDA clusters were identified in 14 (M623, M624, M625, M626, M628, M631, M632, M633, M635, M636, M644, M646, M647, M650) of the 18 genomes, and these genes displayed nearly identical sequential patterns within the BGCs (Figure 4). In addition to the previously known genes (*tdaABCDEF*) involved in TDA biosynthesis, genes responsible for transport and regulation were present (Figure 4). Notably, all TDA BGCs from the 14 strains were located on plasmids rather than on chromosomes. The pattern of one such TDA-containing plasmid of M636 is shown in Figure S2. Seven genes (*cheR*, *cheW*, *cheA*, *cheY*, *cheX*, *mcp*, *cheD*) involved in the process of bacterial chemotaxis were annotated on the plasmid (Figure S2). 

However, key genes (*tdaABCDEF*) related to TDA biosynthesis were also identified in the other four strains (M619, M627, M634, M643), as revealed by BLASTp search (Figure 4). These genes were scattered across different chromosome or plasmid locations. To elaborate, in M619, the *tdaD* gene was found to be located on the chromosome, unlike other strains, where it was found on plasmids (Figure 4). Additionally, a key core gene related to aldehyde dehydrogenase (MaoC like domain) was also located on the chromosome in M643 (Figure 4). Moreover, in M627 and M634, the *tdaF* and *tdaE* genes were separated by nearly 200 genes (Figure 4). 

The different distribution patterns of TDA BGCs indicated gene arrangement variations between different strains, and made us speculate the plausible role of horizontal gene transfer (HGT) in shaping the diversity of this gene cluster. We tested this notion by constructing a phylogenetic tree based on the six key genes (*tdaABCDEF*) involved in TDA biosynthesis, and comparing this with a tree based on single-copy genes. Interestingly, the tree of *tda* genes (Figure 4) exhibited completely different topological structures than the tree based on single-copy genes (Figure S3). For instance, the single-copy gene tree clustered M634 and M636 into the same branch (Figure 2B). The *tda* gene tree, on the other hand, clustered M634 and M636 into different branches with a large distance between them (Figure 4). The different topological structures between the species tree and the *tda* gene tree are indicative of HGT of the *tda* gene cluster.

### 2.4. TDA Production and Antimicrobial Activity

**Figure 5:**
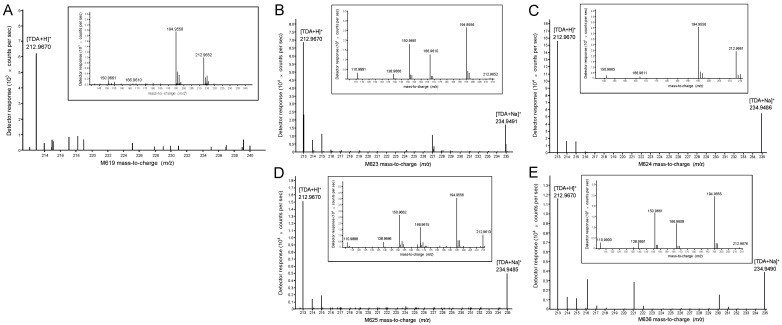
The HPLC-MS/MS detection of TDA production in four *P. inhibens* strains. Two TDA molecules, [TDA + H]^+^ and [TDA + Na]^+^, are detected. The figures in the small boxes: MS/MS fragmentation molecules of TDAs. (**A**) M619; (**B**) M623; (**C**) M624; (**D**) M625; (**E**) M636.

To explore TDA production in *P. inhibens*, five strains (M619, M623, M624, M625, and M636) with a relatively long evolutionary distance of *tda* genes were selected to have their TDA production analyzed using HPLC-MS/MS. These strains were statically cultured at 25 °C for 4 days and then subjected to compound extraction and HPLC-MS/MS. The relative abundances of [M + H]^+^ of TDA in the extracts of these five strains (M619, M623, M624, M625, and M636) were as follows: 6.25 × 10^3^, 6.8 × 10^4^, 1.56 × 10^5^, 1.5 × 10^4^, and 1.16 × 10^4^, respectively, which did not show significant variation (Figure 5). In the chromatogram, the ion at *m/z* 212.967 corresponded to the protonated molecular species [M + H]^+^ of TDA, while the ion at *m/z* 234.949 corresponded to the alkali metal adduct [M + Na]^+^ of TDA (Figure 5). During MS/MS analysis, three to five ion fragments of TDA were detected. The precursor ion of *m/z* 212.967 generated a fragmentation pattern of ions of *m/z* 194.955, 166.961, 150.966, 138.967, and 110.989 (Figure 5). The presence of *m/z* 194.955 probably resulted from dehydration (Figure 5). The subsequent loss of 28 Da corresponded to the neutral loss of a carbon monoxide (CO) molecule, which led to the creation of a tropodithietane cation at *m/z* 166.961 (Figure 5). Further removal of CO from the seven-membered tropone cation resulted in the formation of a six-membered benzo-dithietane cation at *m/z* 138.967 (Figure 5). The subsequent reduction and loss of a hydrogen atom contributed to the formation of a (2-mercapto-2,4-cyclopentadien-1-yl) methyl cation with *m/z* 110.99 (Figure 5). The product ion at *m/z* 151 was previously described in the literature [18] as a collision-induced dissociation fragment (CID) ion of TDA, specifically a tropodithietane cation formed due to the loss of a hydroxyl group.

Considering that all five tested strains produced TDA, as well as the nearly identical structures of the TDAs, we speculated that the five strains may utilize TDA as their major antimicrobial agent. We investigated whether the presumed antibacterial compound TDA can be secreted into the extracellular space and consequently inhibit the growth of other bacteria. We tested the inhibitory effects of the five strains (M619, M623, M624, M625, and M636) on a *V. owensii* strain, ems001, isolated from the intestinal tracts of South American white shrimps with AHPND. 

**Figure 6:**
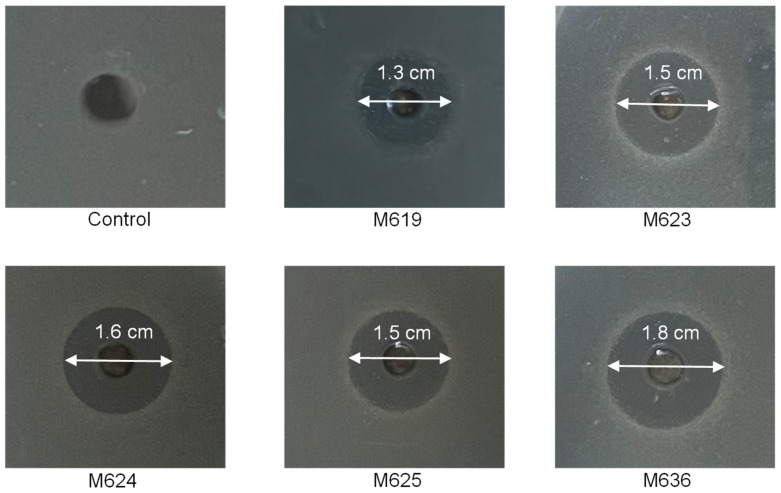
The antibacterial activity of five *P. inhibens* strains against *Vibrio owensii*. The five *P. inhibens* strains were cultured into wells, each carved into the center of an agar plate that was priorly paved with *V. owensii*. Marine Broth 2216 was used as a control.

After co-culturing on agar plates, obvious inhibition zones were observed in the five strains, with diameters ranging from approximately 1.3 to 1.8 cm (Figure 6). Based on the above results, we inferred that *P. inhibens* strains can secrete an antibacterial compound, which is highly likely to be TDA, and this compound exhibits significant antibacterial activity against *V. owensii* ems001.

### 2.5. Distribution of P. inhibens in Marine Biofilms

**Figure 7:**
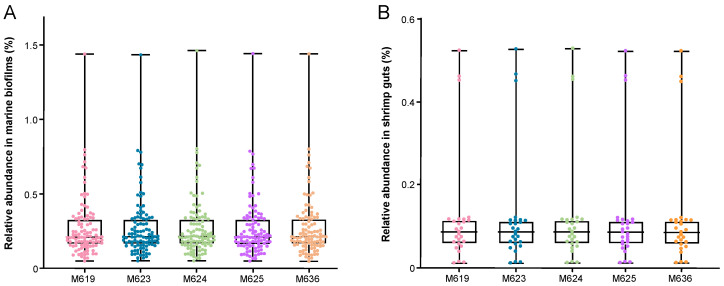
Distribution of *P. inhibens* in both in situ marine biofilms and shrimp guts. (**A**) 101 marine biofilm metagenomes; (**B**) 27 shrimp gut metagenomes.

Finally, to interpret the ecological importance of the *P. inhibens* strains, we analyzed their distribution in both in situ marine biofilm (*n* = 101) and shrimp gut (*n* = 27) metagenomes (Figure 7). The 101 marine biofilm metagenomes were collected across three marine provinces [32]. These were taken from stone and man-made material surfaces [32]. The 27 shrimp gut metagenomes were downloaded from the National Center of Biotechnology Information (NCBI) and the China National Center for Bioinformation (CNCB). The shrimp metagenomes included the microbiota of both larval and adult shrimps’ guts and hepatopancreases. The relative abundance of five *P. inhibens* strains (M619, M623, M624, M625, M636) was indicated by the percentage of metagenomic reads recruited by the five genomes. The distribution patterns of the five strains across the 101 marine biofilms were similar, with ranges of 0.05%–1.44%, 0.05%–1.43%, 0.05%–1.46%, 0.05%–1.44%, and 0.05%–1.44%, respectively, and mean abundances of around 0.27% (Figure 7A). In the 27 shrimp gut metagenomes, their distribution showed ranges of 0.01%–0.52%, 0.01%–0.53%, 0.01%–0.53%, 0.01%–0.52%, and 0.01%–0.52%, with mean abundances of approximately 0.12% (Figure 7B). These findings suggest that *P. inhibens* is broadly distributed in both in situ marine biofilms and shrimp guts.

## 3. Discussion

In the present study, we conducted genomic analyses of 18 *P. inhibens* strains isolated from coastal marine biofilms, evaluated their potential to synthesize secondary metabolites, and studied the diversity and evolution of their respective pathways. Moreover, this study focused on an antimicrobial compound, TDA, and explored its antimicrobial activity against marine *Vibrio* pathogenesis.

Following the suggestion of a previous study that marine biofilms constitute high microbial diversity and functional potential [32], here we employ pan-genome analysis to detect strain-level diversity among *P. inhibens* strains. Each strain harbors considerable proportions of specific functional genes and SNPs, indicating that their strain-specific genetic variations may offer selective advantages in their respective microenvironments. Such adaptation also enhances the evolutionary potential of the population and contributes to the stability of microbial communities in environments subjected to constant challenges [37,38]. One might speculate that efforts towards bacterial isolation from marine biofilms can continuously expand the existing understanding and knowledge of the diversity of marine microorganisms. 

Our analyses of BGCs from *P. inhibens* genomes highlight the potential applicability of this bacterial group as a source of natural products. GCF classification and comparison with previously documented bacterial information underscore the novelty of the BGCs in *P. inhibens* genomes. The RiPPs from these 18 strains are clustered into the same GCF, and the core biosynthetic genes of this RiPP are related to the DUF692 enzyme family. This family includes post-translational modification enzymes involved in RiPP biosynthesis, which can mediate the chemical transformations leading to macrolides and heterocycles [35,36]. However, since the synthesis of RiPPs relies on the collaborative action of multiple enzymes, investigating the specific roles of these enzymes in producing the final products poses significant challenges and may necessitate considerable effort. Moreover, hserlactones are known to be involved in quorum sensing and regulation within microbial communities [39,40]. This functionality may contribute to the survival of *P. inhibens* in marine biofilms.

Phylogenetic trees constructed using single-copy genes and TDA biosynthesis core genes revealed different strain positions, indicating that the whole *tda* cluster had undergone HGT. This observation is consistent with our previous research, which indicated substantial HGT, recombination, and gene losses among BGCs of thalassospiramides [41]. This is also in line with the fact that many TDA BGCs genes are located on plasmids that are transmitted through the gene transfer agent of *Roseobacter* and regulated by the concentration of TDA [42,43]. Moreover, the dispersed distribution of TDA BGCs in four strains indicates recombination of these gene clusters during HGT. Ecologically, TDA may provide competitive advantages to biofilm inhabitants, as it exhibits antimicrobial effects and promotes the formation of a mutualistic relationship [33]. Therefore, biofilm niche adaptation is likely to be a selective force that drives the transfer of TDA BGCs. 

HPLC-MS/MS and antimicrobial experiments performed in the present study revealed the conserved chemical structure, production, and activity of TDAs in different *P. inhibens* strains. Strain M619 only contains six genes (*tdaABCDEF*) for the biosynthesis of TDA. The presence of abundant TDA in the culture product of M619 indicates that these six core genes are enough for the biosynthesis and secretion of this compound. Notably, the *Vibrio* strain used in the antimicrobial experiment was isolated from South American white shrimps that had died from AHPND, a major disease in marine shrimps that can cause severe mortality (up to 100%) within 20–30 days [44]. Several *Vibrio* species such as *V. parahaemolyticus*, *V. punensis*, *V. harveyi*, and *V. owensii* have been identified as causative agents of AHPND in crustacean species [45,46,47]. Moreover, the substantial abundance of *P. inhibens* genomes in marine biofilms and shrimp gut microbiomes suggests their natural inhabitation in these niches. Therefore, *P. inhibens* strains may have significant potential for application as antimicrobial agents for disease control applications in marine aquaculture. 

## 4. Experimental Procedures

### 4.1. Collection of Biofilm Samples and Isolation of Strains

Marine biofilms were scraped off of stones submerged in the subtidal zone of Qingdao (120.145, 39.915), China, with sterile cotton swabs. The biofilm samples were suspended in sterile seawater and promptly transported to the laboratory. For strain isolation, the biofilm bacterial suspension was vortexed and the bacterial cells were resuspended in marine broth 2216E medium (Difco, Franklin Lakes, NJ, USA). The samples were serially diluted to gradients of 10^0^, 10^−1^, 10^−2^, 10^−3^, 10^−4^, and 10^−5^ and inoculated onto 2216E agar plates. The plates were incubated at 25 °C until visible colonies appeared. Individual colonies were then sub-cultured and subjected to polymerase chain reaction (PCR) amplification of the 16S rRNA gene. The PCR products were verified by Sanger sequencing. Preliminary classification of the strains was performed by BLASTn, searching against the 16S rRNA gene sequences in the Nucleotide database of the National Center for Biotechnology Information (Bethesda, MD, USA). TEM of bacterial cells was conducted, as per the method described in our previous study [48].

### 4.2. Whole-Genomic Sequencing and Assembly

For whole-genomic sequencing, the DNA was extracted from strains cultured for 24 h using the TIANamp Genomic DNA Kit (Tiangen, Beijing, China). The obtained genome of each strain was sequenced using the PacBio and Illumina platforms at the Novogene Bioinformatics Institute (Beijing, China). All PacBio reads shorter than 500 bp were filtered out from 1 Gb clean data, and the remaining high-quality reads were assembled using SMRT Link v5.0.1 [49] to produce initial genome assemblies. The obtained assemblies were subsequently corrected using Minimap2 [50] with Illumina reads. All chromosomal sequences were distinguished from plasmids based on the read coverage and circularity of the assembled reads. The completeness and contamination of the genome assemblies were assessed using CheckM [51]. All 18 complete *Phaeobacter inhibens* genome samples were deposited into the China National Center for Bioinformation. The accession numbers are listed in Table S1.

### 4.3. Basic Genomic Information Analyses

In this study, all genomic analyses were performed on a local server installed on the Linux Centos 7 platform. For taxonomic classification, GTDB-Tk (version 0.3.2) [52], which relies on 120 single-copy marker genes, was used. To determine the genomic GC content, Quast (version 5.0.2) was employed [53]. The prediction of tRNA- and rRNA-coding genes was conducted using Prokka (version 1.14.6) [54], while that of ORFs was performed using Prodigal (version 2.60) [55], with only closed-end ORFs returned. The annotation of protein sequences corresponding to all ORFs was performed using BLASTp searches (E-value < 1 × 10^−7^) against the KEGG database (2022 version) sourced from the Kanehisa Laboratory (Japan). Subsequently, the isolated strains were subjected to pangenome analysis using IPGA [56] (https://nmdc.cn/ipga/, accessed on 25 December 2021), which included the analysis of core genes (genes common to all strains), accessory genes (genes present in some strains), and unique genes (genes specific to a single strain).

### 4.4. Analysis of Biosynthesis Potential 

The prediction of BGCs was carried out using the software antiSMASH (version 6.1.1), which was installed on a local Linux platform [57]. The BGCs predicted from the 18 strains and MiBIG (version 3.1) [58] data were clustered based on cosine distance using BiG-SLiCE (version 1.1.0) [59]. A threshold was established where BGCs with a calculated distance greater than 0.2 were classified as distinct GCFs, while those with a distance below 0.2 were grouped into the same GCF. A GCF from the 18 strains was deemed novel if it did not cluster with any GCFs in the MiBIG database, while those that successfully clustered were regarded as not novel. A cumulative bar chart related to BGC distance analysis was plotted using R (version 4.3.2) with the “tidyverse”, “ggsci”, and “cowplot” packages.

### 4.5. Phylogenetic Tree Construction

To construct a phylogenetic tree, all the protein sequences corresponding to *tdaABCDEF* were aligned using the tMuscle algorithm in MEGA (version 7.0.26) [60] as per the following parameters: Gap Open 2.9, Gap Extension 0, and Hydrophobicity Multiplier1.2. Clustering was performed by the unweighted pair group method with arithmetic mean and a minimum diagonal length of 24. The alignments were processed using Gblocks (version 0.91) [61] that eliminated less informative sites. A maximum likelihood phylogenetic tree was built using the Jones–Taylor–Thornton matrix-based model, with 500 bootstrap replicates for robustness assessment. The single-copy gene tree was built using seven genes, including *dnaG*, *nusA*, *pgk*, *pyrG*, *rplB*, *rpoB*, and *tsf*, following the same method as the *tda* gene tree.

### 4.6. HPLC-MS/MS Analyses

Five strains (M619, M623, M624, M625, M636) were statically incubated at 25 °C for 4 days and then the cultures were transferred to a 250 mL Erlenmeyer flask. Each flask was mixed with an equal volume of ethyl acetate acidified with formic acid (0.1%) and ultrasonicated for 15 min. The extraction process was carried out thrice with acidified ethyl acetate. The organic phase was retained and concentrated under reduced pressure. The concentrated organic phase was dissolved in 2 mL of methanol by ultrasonication and then subjected to centrifugation at 8000 g for 5 min. The supernatant was filtered through a 0.22-µm organic nylon membrane (Millipore, Boston, MA, USA) to obtain the final filtrate.

HPLC-MS/MS was performed using an Agilent 6530 LC/Q-TOF (Agilent, Santa Clara, CA, USA) coupled to an Agilent 1290 ultra-high-pressure liquid chromatography instrument (UHPLC; Agilent, USA). The separation of compounds was carried out with a Waters ACQUITY UPLC HSS T3 column (2.1 × 100 mm, 1.8 μm particle size, Waters, Milford, MA, USA) using methanol (solvent A) and pure water (solvent B). The column temperature was maintained at 35 °C and a gradient elution was applied at a flow rate of 0.3 mL/min for 32 min, starting from 10% solvent A, gradually increasing to 90%, then decreasing back to 10% from 25.5 to 32 min. The injection volume was 1 µL. The electrospray ionization mass spectral data were recorded in both positive and negative modes, with a mass range of *m/z* 100 to 1700. The capillary voltage and cone voltage were set at 4 kV and 100 V, respectively. The sheath gas temperature and flow rate were maintained at 350 °C and 11 L/min, respectively, while the drying gas temperature and flow rate were 300 °C and 5 L/min, respectively. CID of the analytes was achieved using collision energies of 10, 20, 40, and 60 eV, with argon as the collision gas. 

The collected raw data were imported into the B.08.00 Agilent Masshunter Qualitative Analysis software. For all unknown compounds, an identification workflow was established using the wizard settings and method templates. Protonated molecules [M + H]^+^ and alkali metal adducts [M + Na]^+^ were detected using the positive-ion mode in the full-scan mass spectra. Later, these unique compounds were characterized using accurate mass measurements of precursor ions and their corresponding mass measurements, as well as tandem mass spectrometric fragment ions. Their identities were further confirmed by determining their accurate mass and CID spectra.

### 4.7. Antibacterial Test of Phaeobacter Inhibens Against V. owensii

The strain *V. owensii* ems001 was isolated from the intestinal tracts of South American white shrimps, which were sampled during an AHPND event at a fish farm in Cangzhou, Hebei, China. Five strains of *P. inhibens* (M619, M623, M624, M625, M636) were statically incubated in 2216 marine broth medium at 25 °C for 4 days. The 2216 agar medium was sterilized and cooled to 45–50 °C, then mixed with *V. owensii* ems001 culture at a volume ratio of 1:100. A sterile well was made using a sterile puncher in the center of each of the 2216 solid agar plates containing *V. owensii* ems001. *P. inhibens* cultures were added to the wells in the treatment group at 80 µL per well, while samples of 2216 marine broth of the same volume were added to the control wells. After 24 h, inhibition zones were observed and measured.

### 4.8. Abundance Analysis of P. inhibens in Marine Biofilm and Shrimp Gut Metagenomes

The original metagenomic data were taken from previous studies [32]. Reads were filtered using the NGS QC Toolkit software (version 2.0) to remove low-quality sequences (quality score < 20) [62]. Subsequently, the FastqToFasta module included in the software was employed to convert the quality-controlled metagenomic data into the FASTA format. SeqKit software (version 2.8.2) was utilized to randomly sample one million sequences to construct the metagenomic database [63]. The genomes of five *P. inhibens* strains (M619, M623, M624, M625, M636) were then aligned with the metagenomic database using BBMap (version 35.85) [64], and the abundance of each strain was calculated based on the number of matched reads in the metagenomic datasets including 101 marine biofilms and 27 shrimp guts. The metagenomic data information for the marine biofilms and shrimp guts can be found in Table S3.

## 5. Conclusions

By focusing on *P. inhibens* strains, this study sheds light on the genomic features and biosynthetic potential of marine biofilm bacteria. In particular, the findings highlight the role of HGT in shaping the evolution of TDA BGCs, plausibly in a biofilm niche-specific manner. While the TDA BGCs are rather diverse, all the examined strains produce TDA as their major secondary metabolite. The confirmation of their strong antibacterial activity against the pathogenic *Vibrio* strain, as well as the wide distribution of *P. inhibens* in natural marine biofilms and shrimp guts, underscore a promising new application of these strains to control the infections caused by marine pathogens of shrimps. Subsequent research will focus on integrating genomics, metabolomics, and animal experiments for the in-depth exploration of pharmaceutical resources from marine biofilm-associated bacteria. 

## Data Availability

The authors declare that all relevant data supporting the findings of this study are available within the article and its Supplementary Material files. The complete genomes of 18 *Phaeobacter inhibens* strains were deposited in the China National Center for Bioinformation (CNCB) database under the accession numbers shown in Table S1.

## Supplementary Materials

The following supporting information can be downloaded at: https://www.mdpi.com/article/10.3390/md22110492/s1, Figure S1. Transmission electron microscopy observation of a representative strain, *Phaeobacter inhibens* M619; Figure S2. A representative plasmid containing the tropodithietic acid (TDA) biosynthetic gene clusters; Figure S3. A maximum likelihood phylogenetic tree constructed based on single-copy genes; Table S1: Overall information of the *Phaeobacter inhibens* genomes; Table S2: Functional gene annotation of the *P. inhibens* genomes; Table S3: Metagenomes of 101 marine biofilms and 27 shrimp guts.
