# Supplementary material for: Genomic Features and Antimicrobial Activity of Phaeobacter inhibens Strains from Marine Biofilms
Source: Mar Drugs. 2024 Oct 31;22(11):492. doi: 10.3390/md22110492 (PMC11595833; doi:10.3390/md22110492)
Supplement: Supplementary file 1 [file marinedrugs-22-00492-s001.zip › FigureS1_S3.pdf]

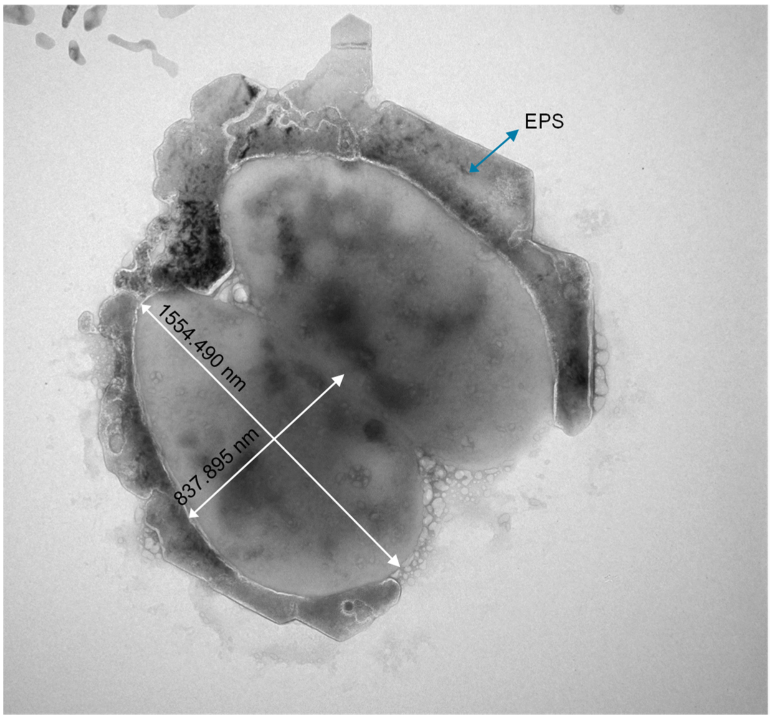

Figure S1. Transmission electron microscopy observation of a representative strain, *Phaeobacter inhibens* M619. The white arrows indicate cell size and the blue arrow indicates extracellular polymeric substance (EPS).

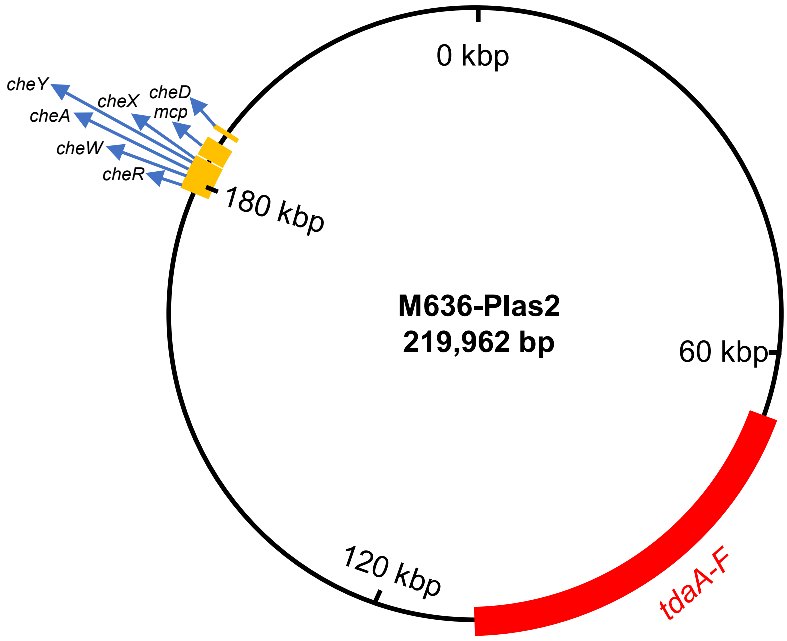

Figure S2. A representative plasmid containing the tropodithietic acid (TDA) biosynthetic gene clusters. This plasmid is derived from the strain M636.

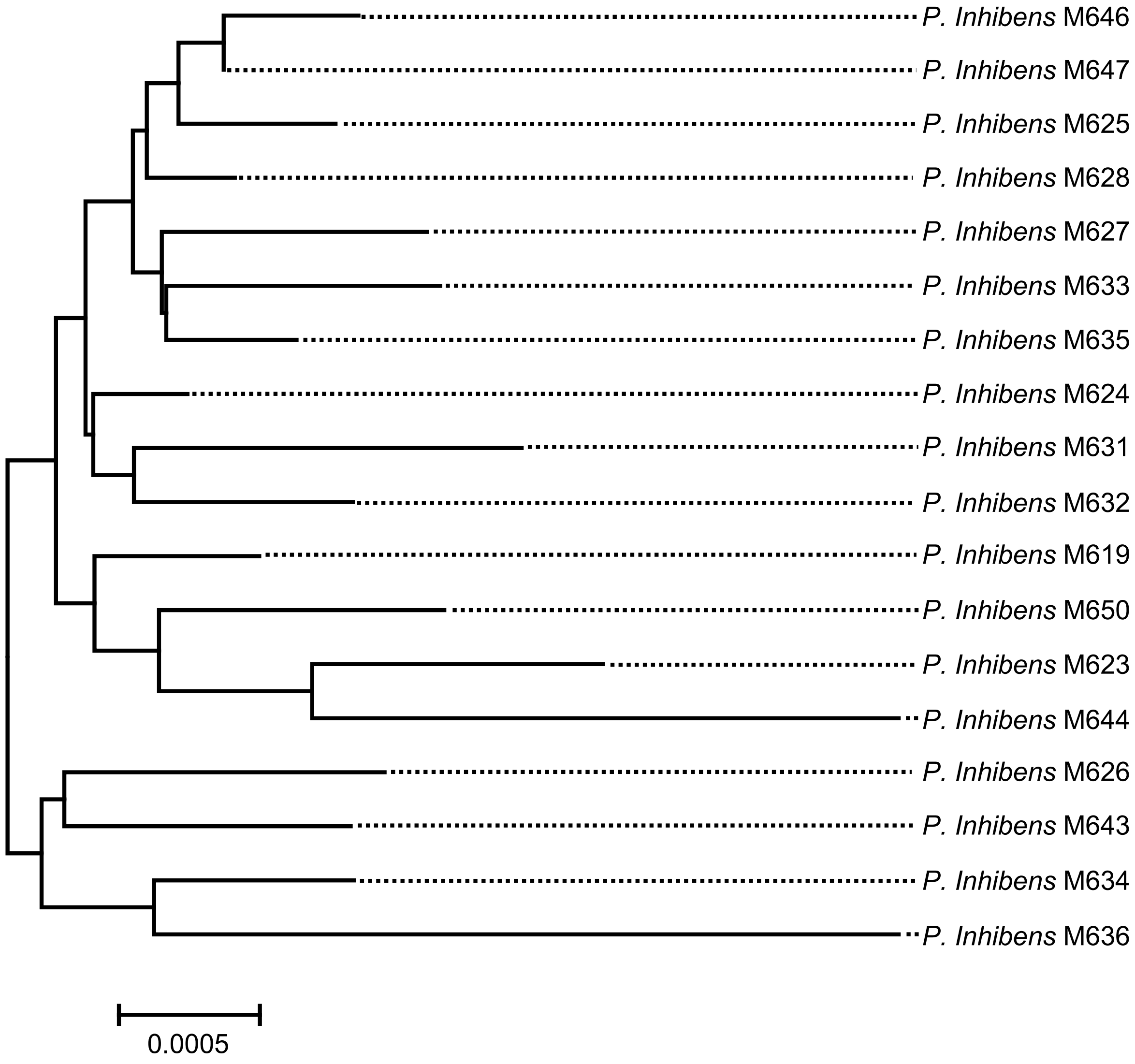

Figure S3. A maximum likelihood phylogenetic tree constructed based on single copy genes. Seven genes, including *dnaG*, *nusA*, *pgk*, *pyrG*, *rplB*, *rpoB*, and *tsf* were extracted from the 18 *P. inhibens* strains and used to build the tree.
